# Deficiency of adaptive immunity does not interfere with Wallerian degeneration

**DOI:** 10.1371/journal.pone.0177070

**Published:** 2017-05-05

**Authors:** Christopher R. Cashman, Ahmet Hoke

**Affiliations:** 1 MSTP/MD-PhD Program, Johns Hopkins School of Medicine, Baltimore, Maryland, United States of America; 2 Department of Neuroscience, Johns Hopkins School of Medicine, Baltimore, Maryland, United States of America; 3 Department of Neurology, Johns Hopkins School of Medicine, Baltimore, Maryland, United States of America; Szegedi Tudomanyegyetem, HUNGARY

## Abstract

Following injury, distal axons undergo the process of Wallerian degeneration, and then cell debris is cleared to create a permissive environment for axon regeneration. The innate and adaptive immune systems are believed to be critical for facilitating the clearance of myelin and axonal debris during this process. However, immunodeficient animal models are regularly used in transplantation studies investigating cell therapies to modulate the degenerative/regenerative response. Given the importance of the immune system in preparing a permissive environment for regeneration by clearing debris, animals lacking, in part or in full, a functional immune system may have an impaired ability to regenerate due to poor myelin clearance, and may, thus, be poor hosts to study modulators of regeneration and degeneration. To study this hypothesis, three different mouse models with impaired adaptive immunity were compared to wild type animals in their ability to degenerate axons and clear myelin debris one week following sciatic nerve transection. Immunofluorescent staining for axons and quantitation of axon density with nerve histomorphometry of the distal stump showed no consistent discrepancy between immunodeficient and wild type animals, suggesting axons tended to degenerate equally between the two groups. Debris clearance was assessed by macrophage density and relative myelin basic protein expression within the denervated nerve stump, and no consistent impairment of debris clearance was found. These data suggested deficiency of the adaptive immune system does not have a substantial effect on axon degeneration one week following axonal injury.

## Introduction

Following an acute injury, such as a transection, the distal aspect of axons actively degenerate, and their debris is cleared in a process known as Wallerian degeneration [1]. The immune system is a critical component of axon degeneration and regeneration and consists of both an innate and adaptive subsystem. The innate immune system describes the rapid-response, “hard wired” phagocytic cells often responsible for bacterial and debris clearance, while the adaptive immune system is slower, but more flexible, specific, long term, and often organizes the innate response. The innate immune system includes macrophages, granulocytes, and complement, while the adaptive immune system contains lymphocytes including B- and T-cells (as reviewed in [2]). Antibodies and interleukins bridge these two sides of the immune system where antibodies from B-cells can coat, “opsonize,” antigens to facilitate phagocytosis by the innate and adaptive immune system [3,4], and interleukins released from one side of the immune system can both activate and attenuate additional cell recruitment or activation on the same or other side (as reviewed in [5]). Complement, the protease cascade responsible for recruitment, opsonization, and, occasionally, direct attack of pathogens, also bridges the innate and adaptive immune system [6,7].

Within this complex ballet of inter- and intracellular signaling, the immune response has been found to be essential for proper degeneration of the peripheral nervous system, which predicates successful regeneration. While the improper development of a response against self-antigens can lead to cytotoxic T-cell mediated axon degeneration in a model of multiple sclerosis [8], in the peripheral nervous system, autoantibodies may, in fact, facilitate myelin clearance [9].

Additionally, immune surveillance and debris clearance are facilitated by complement, the components of which are synthesized by Schwann cells, whereby it may coat membrane debris and attract macrophages (as reviewed in [10,11]) to the site of injury. Macrophages are critical for proper phagocytosis and clearance of debris to allow axon regeneration [12,13]. Complement also functions to reduce autoimmunity by clearing apoptotic cells [3].

The importance of the adaptive and innate immune systems in degeneration is largely due to their ability to facilitate myelin debris clearance. Myelin clearance is essential for proper regeneration in the peripheral nervous system and is one of the determinants of successful regeneration in the peripheral versus central nervous system (as reviewed in [14]), so it follows that impairments in the complement, innate, or adaptive branches of the immune system may lead to aberrant degeneration and, thus, regeneration. More specifically, while many studies have focused on the harm of deficiency of the adaptive immune system on motor neuron survival [15–19] in certain strains of mice [20] where T-cells [21] help support motor neurons by release of neurotrophic factors [22], little work has examined the effect of adaptive immunodeficiency on peripheral axon degeneration where clearance of debris is necessary for proper regeneration. Given the difference of the peripheral nervous system versus the central nervous system, i.e the lack of microglia in the periphery and more successful regeneration in the periphery following myelin clearance, immunodeficiency may have a fundamentally different effect on the neuronal axon in the peripheral nerve than the neuron cell body in the central nervous system, and has, thus far, been understudied. Previous work has demonstrated the critical role of the innate immune system as an effector of debris clearance (as reviewed in [12]), but thus far the role of the adaptive immune system in the regulation of the response remains unclear. More specifically, while the innate immune system is necessary for myelin clearance, its sufficiency for this purpose, in the setting of adaptive immune system deficiency, has not thus far been extensively investigated. Additionally, debris clearance in two stages, with activated Schwann cells first clearing myelin immediately following injury, then macrophages, as components of the innate immune system, entering the tissue to ultimately “take over” the response [23]. The effect of deficiency of the adaptive immune system on peripheral nerve degeneration is studied herein.

A number of immunodeficient rodent models, including “SCID” [24] and “Rag1” mice [25], exist that lack the ability to produce functional antibodies and T-cell receptors due to deficiency in V(D)J recombination. A different model lacks mature T-cells due to incomplete thymus development, the “RNU” or “nude” mouse [26]. These models will be used to determine the effect of immunodeficiency on axonal degeneration in the peripheral nerve following transection.

## Results and discussion

Given previous work showing the importance of autoantibodies and the adaptive immune system for clearance of myelin and axonal debris [9], it may be expected to see deficiencies in degeneration in animals with impaired adaptive immunity despite a preserved innate response. To test this hypothesis, transection of the sciatic nerve in three different adaptive immune system deficient mouse models and one healthy control, all of them C57BL/6 strain (Table 1), were performed at six months of age, and axonal degeneration and myelin clearance assayed one week later. It should be noted that among these models, there may be variable antibody titers. A single time point was chosen to examine the terminal effect of adaptive immunodeficiency, as debris clearance by the innate immune system is well under way by one week of denervation [27], so any significant delay in axon degeneration should be appreciated at this time point. Investigation of changes over time were deemed to be outside of the scope of this study.

**Table 1:** Animal models of immunodeficiency.

*Genotype*	*Abbreviation*	*Deficiency*	*Affected lineage*
Wild type	WT	None	All lineages intact
Prkdc^scid^/Prkdc^scid^ (SCID)	Prkdc^-/-^	V(D)J recombination/DNA synthesis	Immature and mature B and T cell (adaptive)*; Intact innate
Rag1^tm1Mom/tm1Mom^	Rag^-/-^	V(D)J recombination	Immature and mature B and T cell (adaptive); Intact innate
Foxn1^nu/nu^ (nude)	Foxn1^-/-^	Thymus development	Mature T Cells; Intact innate

* Some SCID mice may begin to develop detectable levels of serum immunoglobulins, suggestive of an escape from immunodeficiency.

**Fig 1:**
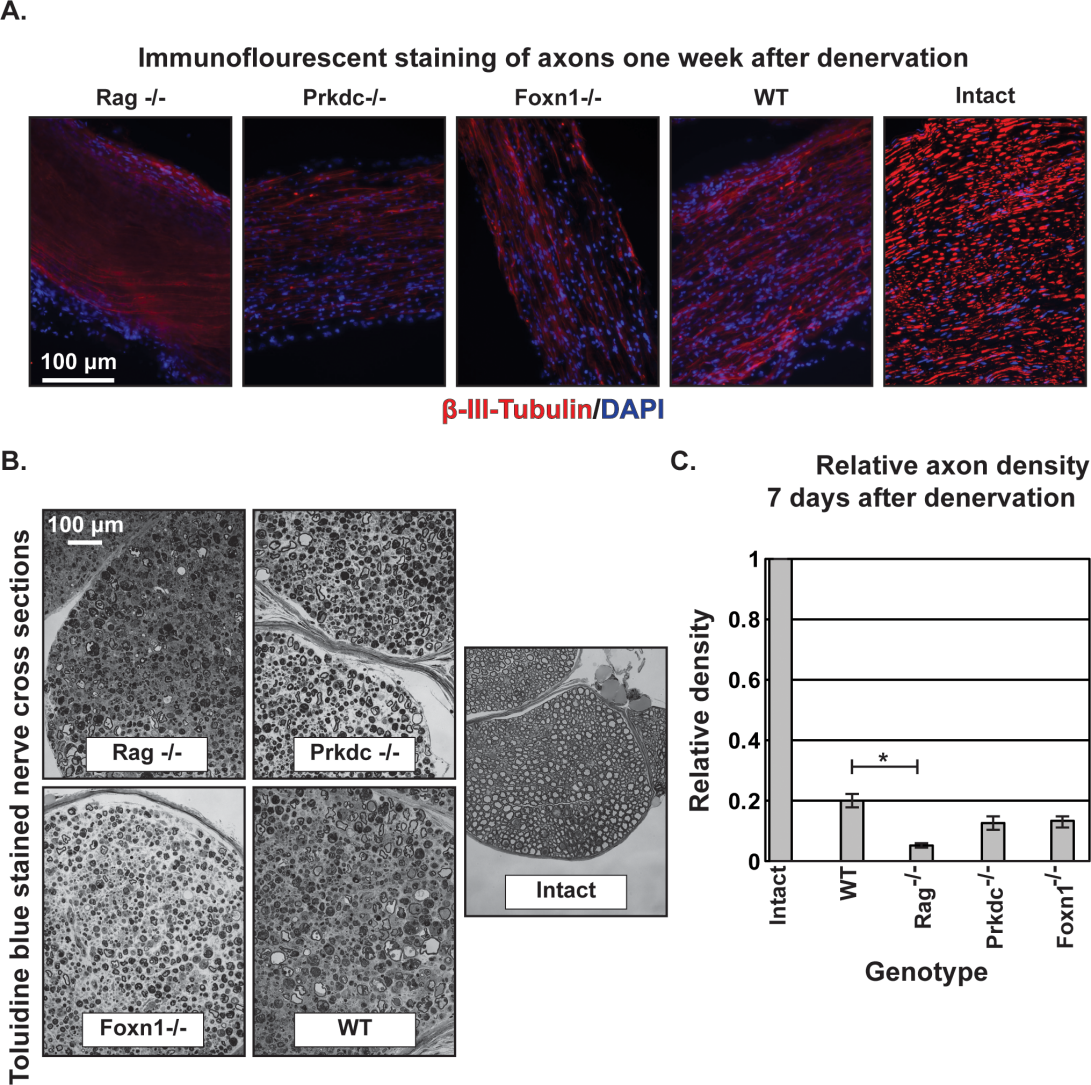
Immunodeficency and degeneration. A. Axonal staining in widefield epifluorescent images (20x) of longitudinal sections of one-week denervated sciatic nerves. Intact nerve is not denervated. Red: α-β-III-tubulin; Blue: DAPI. B. Transmitted light images (20x) of thick plastic sections of toluidine blue stained sciatic nerve cross sections. C. Quantification of the axon density in the denervated tibial nerve in comparison to an intact nerve in the images in the toluidine blue stained sections. Error bars are SEM. * = p<0.05 by ANOVA with Tukey’s post-hoc analysis, α = 0.05. n = 3 animals for WT, Rag^-/-^, Foxn1^-/-^, and n = 2 for Prkdc^-/-^. Intact nerve is included for relative comparison only, with only one sample represented. Statistical comparison to the intact nerve by 95% confidence interval with a Student’s t-distribution, α = 0.05.

To reduce the likelihood of proximal and distal stump reconnection, a rigorous denervation protocol was performed whereby the sciatic nerve was triple ligated, transected, and the proximal stump retroflected and sutured to adjacent muscle. One week after denervation, the distal sciatic nerve stumps were immunostained for β-III-tubulin, where, like denervated wild type controls, no intact axons were observed in any of the immunodeficient genotypes (Fig 1A). These data were supported by toluidine blue stained distal sciatic nerve cross sections, where few axons, but many cells, were visible (Fig 1B). Quantitation of the images showed no statistical difference among the immunodeficient animals, although Rag^-/-^ animals tended to have a lower density of intact axons such that it was significantly lower than the density in the denervated wild type nerve, while the Prkdc^-/-^ and SCID genotypes were no different from wild type (Fig 1C). It should be noted that Prkdc^-/-^, like Rag^-/-^, animals lack both B- and T-cells, but are generally considered more “leaky” with eventual appearance of immunoglobulins in the periphery, especially in older animals, with ~20% of seven to eight month old animals positive for peripheral immunoglobulin [28]. This leakiness may explain their failure to display the same phenotype as the “cleaner” Rag^-/-^ animals.

Given the axon density data, with the exception of the Rag^-/-^ genotype, impaired adaptive immunity does not remarkably affect axonal degeneration. In fact, adaptive immunodeficiency may increase axonal degeneration, as evidenced by the lower density of axons in the Rag^-/-^ animals. These data agreed with recent work showing greater axonal degeneration in Rag^-/-^ mice in a model of dysmyelination due to mutations in myelin basic protein [29]. In the study by Berghoff, et al., macrophages were retained in the nerve for longer periods than immunocompetent animals, but myelin disruption was not significantly different between the immunocompetent and immunodeficient dysmyelinated animals [29]. These data suggested complete B- and T-cell loss reduced axonal protection, perhaps from overactive macrophages that could not be inactivated by helper T-cells, in a manner separate from myelin debris clearance [29]. The unusual separation between these two processes, when autoantibodies and macrophages are usually critical for myelin clearance and regeneration [9,13], motivated study of macrophage numbers and myelin clearance in the denervated immunodeficient mice. Notably, the particular immunodeficient mouse strain used in the aforementioned Vargas, et. al study were a B-cell only knockout, JHD mice, so a direct comparison to the strains use in this study may be difficult due to the possibility of variable antibody titers, but the general pattern of adaptive immunity is still relevant.

**Fig 2:**
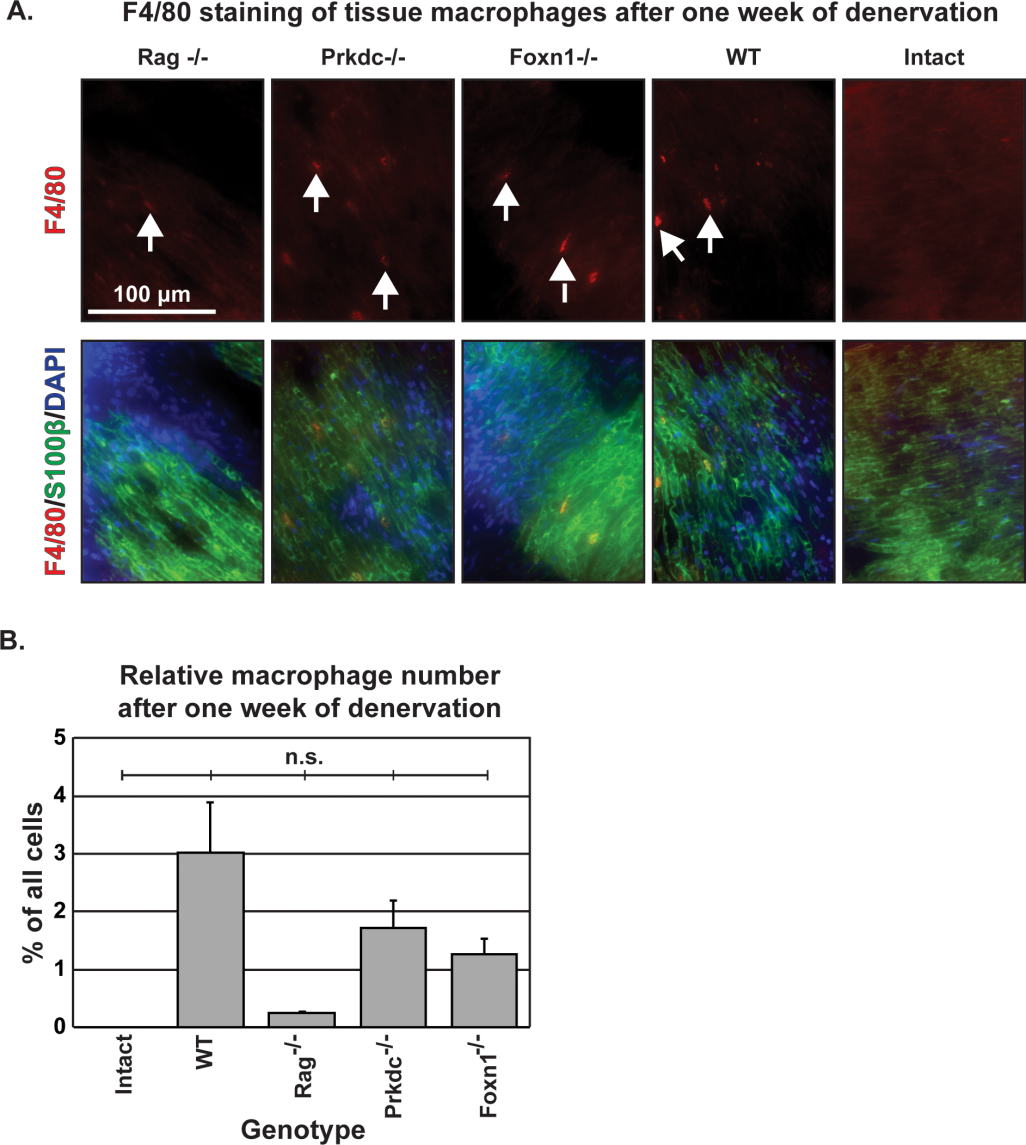
Macrophage infiltration. A. Macrophage staining in widefield epifluorescent images (43x) of longitudinal sections of one-week denervated sciatic nerves in immunodeficient and wild type control animals. Intact nerve was not denervated. Top row: F4/80 (macrophage; red) staining only. Arrows highlight F4/80 positive cells. Second row: Macrophage staining merged with Schwann cell staining (α-S100β; green) and nuclear labeling with DAPI (blue). B. Quantitation of macrophages per high powered field, relative to total cell number. No significant difference among the five groups by ANOVA. n = 3 animals for Rag^-/-^, Foxn1^-/-^, WT, n = 2 animals for Prkdc^-/-^. n = 1 animal for intact (for visual comparison only). Error bars are SEM.

Given the lack of autoantibodies in the immunodeficient mice, fewer macrophages would be predicted to be present in the tissue, and, as a result, less myelin cleared after one week of denervation. Alternatively, there could be more macrophages, as observed in the aforementioned model of dysmyelination [29]. Regardless, the immunodeficient models would be expected to have a different density of macrophages than the wild type. Myelin clearance in immunodeficient animals was comparable to wild type animals, as assayed by macrophage number and relative levels of myelin basic protein. The macrophage marker F4/80 was used to stain longitudinal sections of the one-week denervated sciatic nerve. Macrophages were visible in all sections (white arrows) and did not visibly co-localize with the Schwann cell marker S100β (Fig 2A). Quantitation confirmed the observed pattern (Fig 2B), with no statistically significant difference among the denervated groups, although immunodeficient animals did show a trend toward fewer macrophages, with Rag^-/-^ animals with the lowest density. Thus, immunodeficiency may lead to decreased macrophage invasion, but is subtle, and the data may be underpowered to detect a difference. Nonetheless, the lowest density of macrophages aligned with the most complete model of immunodeficiency, which supports the original hypothesis of immunodeficiency impairing degeneration. This pattern does conflict, however, with the study by Berghoff and colleagues, as they described a higher density of macrophages in Rag^-/-^ immunodeficient animals, although this may be a result of model (dysmyelination versus degeneration) and age difference. However, this finding suggests the trend toward decreased axon density in Rag^-/-^ is not dependent on macrophage number.

**Fig 3:**
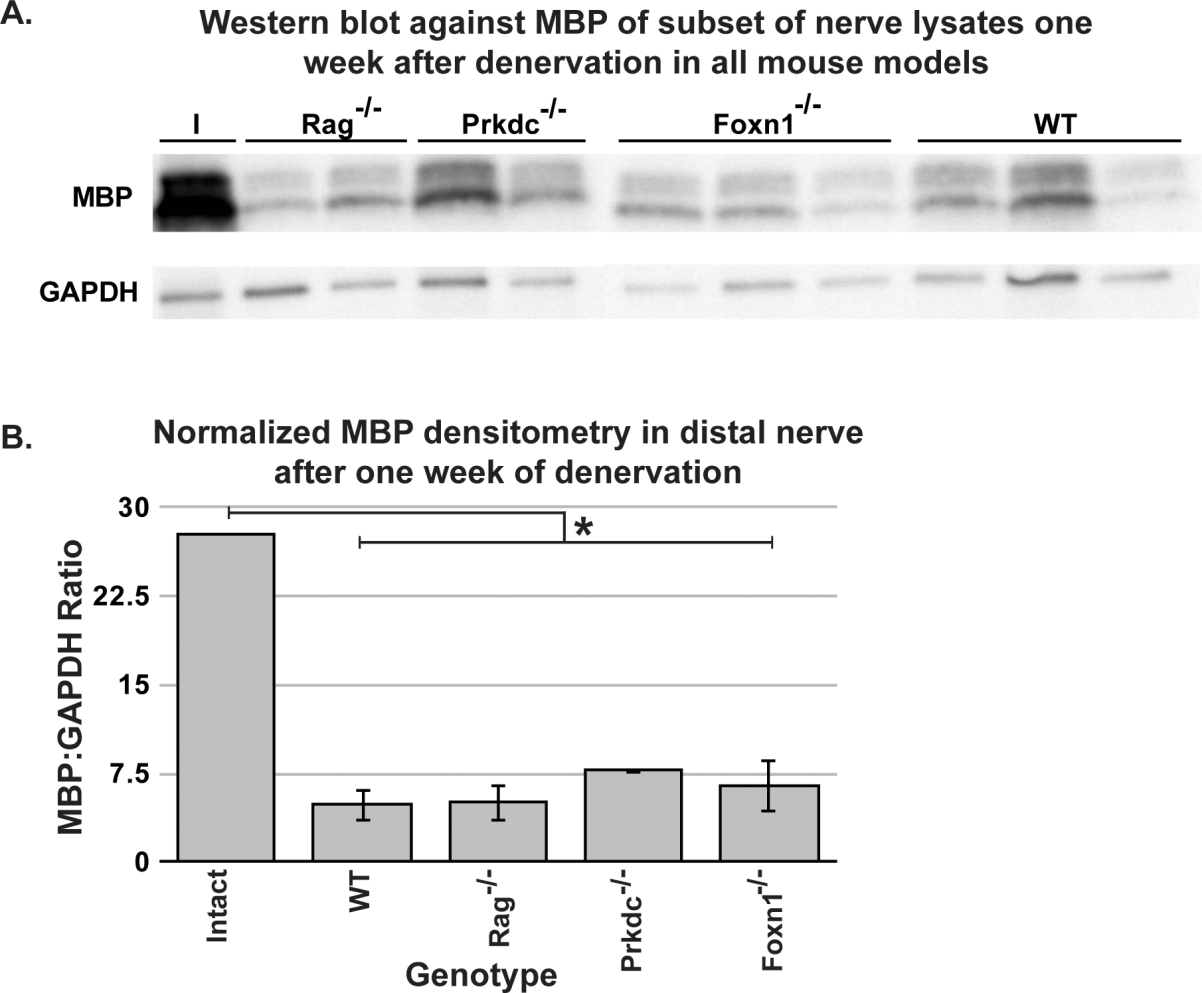
Clearance of myelin basic protein. A. Western blot of myelin basic protein and GAPDH of representative distal sciatic nerve lysates. Each column represents an animal. I = intact nerve B. Ratio of MBP to GAPDH density from the Western blot in A. Note that the intact ratio is for scale only, since it is not replicated. n.s = not significant (p>0.05 by ANOVA). Error bars are SEM. n = 3, 2, 3, 3 animals for Rag^-/-^, Prkdc^-/-^, Foxn1^-/-^, wild type.

More importantly, the ultimate role of the immune system is to clear myelin, so this activity in the setting of adaptive immune system deficiency was also assayed, effectively determining innate immune system sufficiency for myelin clearance. A Western blot of samples of the distal nerve lysates against myelin basic protein (MBP), one of the major proteins in peripheral nervous system myelin [30] (as reviewed in [31]), and GAPDH showed myelin basic protein (MBP) bands significantly less dense than an intact nerve but not consistently different than a denervated wild type nerve (Fig 3A). Quantitation of the ratio of MBP to GAPDH band densities confirmed the initial observations, with no difference in ratio among all denervated genotypes but a significant difference from an intact nerve by 95% confidence interval (Fig 3B). This finding is critical since one of the major roles of macrophages in degeneration is myelin clearance (as reviewed in [12]), and a failure to detect a phenotype of myelin clearance and axonal degeneration strongly disproves the hypothesis that adaptive immunodeficiency leads to a significant impairment of axonal degeneration/debris clearance in the peripheral nervous system. The failure to see any substantial degenerative phenotype in animals with no adaptive immune system may be the result of an intact complement cascade, demonstrating the sufficiency of the innate immune system to effect adequate myelin clearance seven days after injury. While the adaptive immune system can modulate complement, complement also functions independently to clear axonal and myelin debris via the membrane attack complex (as reviewed in [10]). In the absence of immunoglobulins and functional T-cells, complement or increased Schwann cell myelinophagy may compensate for this deficiency to effect nearly normal debris clearance by one week of denervation (reviewed in [10,32]).

## Conclusions

Careful testing of three different mouse models of adaptive immune system deficiency showed immunodeficiency had no significant impact on debris clearance in axonal degeneration at one week of denervation. No major defects in axonal degeneration, macrophage infiltration, or myelin degradation were observed at this time point. While the Rag^-/-^ mice, the most complete model of immunodeficiency used, did have significantly lower axon density than wild type animals, the opposite relationship was predicted by the original observation and hypothesis. Other laboratories have observed increased axonal degeneration in Rag^-/-^ animals associated with an increased macrophage density, as well [29]. The non-significant difference in macrophage number, with a trend toward fewer macrophages, suggests the decreased axon density in Rag^-/-^ animals is not due to increased macrophage activity at one week of denervation, but does not exclude the possibility of different kinetics of myelin clearance, although the more mature state of denervation in the Rag^-/-^ model is still surprising. Rather, perhaps the decreased axon density and macrophage number in the Rag^-/-^ animas is representative of a more rapid or efficient innate immune response in clearing the debris, where by one week following denervation, debris clearance has completed and the immune response has begun to decrease. Additional studies in the time course of degeneration would help address this outstanding question. Certainly, these data also refute the hypothesis that immunodeficiency of the adaptive immune system would impede degeneration. Finally, myelin clearance was preserved across all genotypes. While the latter finding may be a result of a relatively intact complement system in these immunodeficient models (which have a functional innate immune response), additional experimentation on Rag^-/-^ animals to determine the mechanism of the subtly increased nerve degeneration is necessary. Additionally, it is possible that earlier time points would be more sensitive to impaired degeneration as the axon fragments and myelin break down within the first week (summarized in [10]). Nonetheless, by one week of denervation, when the innate immune response, especially macrophages [23], begins to dominate the debris clearance process, the effect of a disordered adaptive immune system on clearance is minimal.

Thus, immunodeficiency of the adaptive immune system does not lead to impaired degeneration one week after injury and may, in some instances, may increase debris clearance. These data showed that the innate immune system is able to effect an adequate response for myelin clearance by one week of denervation. While the exact effect of adaptive immune deficiency on the kinetics of myelin clearance were not examined in this study, it is clear that by one week of denervation, when the innate immune response begins to dominate the clearance process, the adaptive immune system is, and was, unnecessary. Despite the final conclusion of this study ultimately being negative, the observations made herein are still interesting and unexpected. Indeed, while the immune system is certainly important for degeneration and regeneration, the exact role in debris clearance of each arm (complement, innate, adaptive) and cell type (macrophages, B-cells, helper T-cells, cytotoxic T-cells), and how they interact and compensate for the other, are still under investigation. In this case, the finding that deficiency in the adaptive immune system does not, on its own, lead to defects in degeneration in a transection injury model is valuable. Furthermore, the effect of disruption of the adaptive immune system on regenerative potential should be investigated, to determine the full effect, if any, of adaptive immunodeficiency on axonal injury and repair. Additionally, it is reassuring that modification of the immune system, such as immunosuppression by cyclosporine or immunodeficiency, does not appreciably affect host degeneration, and affirms their value in transplant experiments.

## Materials and methods

### Animals

All animal surgeries and euthanasia were conducted under protocols approved by the Johns Hopkins University Animal Care and Use Committee following the guidelines established by the National Institutes of Health and the American Association for the Accreditation of Laboratory Animal Care. All animals were housed under standard conditions. Mice were acquired from Jackson Laboratory (C57Bl6 wildtype: strain 000664, SCID: strain 001913; Rag^-/-^: strain 002216; Nude: strain 00819). All animals were sacrificed by isoflurane anesthesia followed by carbon dioxide euthanasia, in accordance with institutional guidelines.

### Surgical denervation

For all surgeries, anesthesia was induced with 2.0–2.5% isoflurane/oxygen (Piramal Healthcare). Seven to eight six-month old mice of all four genotypes were denervated, as follows. The biceps femoris was exposed following a 1.5 cm incision from the sciatic notch along the midline of the left upper hind limb after shaving and chemical depilation (Nair). The muscle’s insertion to the femur was transected and traction applied to display the sciatic nerve and posterior femoral cutaneous nerve. Connective tissue around the sciatic nerve was dissected with spring scissors and 5–0 forceps (Fine Scientific Tools). Three ligatures were applied with 4–0 nylon sutures around the proximal most aspect of the sciatic nerve. The nerve was then transected between the first and second ligatures. The proximal aspect of the distal stump was loosely sutured to the adductor magnus with an extra loop from the penultimate distal ligature, while the distal aspect of the proximal stump was reflected proximally and buried into the adjacent biceps femoris using two to three 10–0 polyamide or nylon sutures. After denervation, the muscle and skin were returned to their normal position and the incision closed with surgical staples. A triple antibiotic ointment (Neomycin/Polymyxin/Bacitracin ophthalmic ointment, Akron) and spray on a bandage (“New Skin,” Medtek) were applied, in that order, to the incision site after closure. Staples were left in place until samples were collected one week later.

### Tissue fixation, electron microscopy and immunostaining

Nerve samples were collected from half of all denervated animals for staining, while the other half were used for protein extraction. Samples for staining were divided for histomorphometry and immunofluorescent staining.

Samples for nerve histomorphometry were embedded in Epson resin, and cut along the long axis into semi-thin sections of approximately 1 μm, and stained with 1% toluidine blue/1% sodium tetraborate by the Johns Hopkins Neurology electron microscopy core.

For immunofluorescent staining of tissues, the remaining nerve samples were fixed overnight at 4°C in 4% paraformaldehyde in PBS. After fixation, samples were transferred to 15% sucrose (Sigma-Aldrich, S5015) in PBS for 24 hours, then a 30% sucrose solution for an additional day. Samples were stored at 4°C prior to embedding for cryosectioning. Tissue samples were embedded in OCT cryoprotectant (TissueTek, 4583) and frozen at -80 for at least one day. Samples were then longitudinally sectioned on a cryostat (Microm HM 505E) at 10–12 μm sections. Glass mounted tissue sections represented consecutive sections. Samples were stored at 4°C until use.

For immunostaining, sectioned tissue samples were washed three times in PBS then permeabilized for 10–15 minutes with 0.1% Triton X-100 (Sigma-Aldrich, X-100). The samples were washed again and blocked for 30–60 minutes at room temperature in a buffer containing 5% normal goat or donkey serum (Cell Signaling Technology, 5425S or Sigma, D9663, respectively), 0.5% Tween 20 (MP Biomedicals, Tween201) in PBS. Primary antibodies were diluted in blocking buffer and incubated with samples overnight at 4°C. The next day, samples were thoroughly washed with PBS. Secondary antibodies were then prepared in blocking buffer and incubated with samples for one hour at room temperature. All samples were then mounted with mounting solution containing the nuclear stain DAPI (Vector H-1200) and sealed with nail polish. Note that all secondary antibodies were also validated against no primary controls to assess background activity

Axons were stained with mouse α-β-III-tubulin (1:1000 Promega, G7121) with secondary antibody goat α-mouse IgG1, rhodamine (1:200 of 1:1 glycerol to antibody solution; Jackson Immuno, 115-295-205). Macrophages were stained with primary antibody rat α-F4/80 (1:100, BioRad, MCA497GA) and secondary antibody goat α-rat, AF546 (1:400; Life Technologies, A11081). Schwann cells were stained with primary antibody rabbit α-S100β (1:400; Dako, Z0311) and secondary goat α-rabbit, FITC (1:400; Vector, GI-1000).

Macrophages were counted in 43x images of stained sections with an average of six images per animal. Macrophage number was normalized to total cell number visualized by DAPI. These average values were combined with animals of the same genotype for final mean values.

### Imaging

Widefield epifluorescent images were obtained on an Olympus IX51 inverted microscope with a mercury bulb (Olympus, BH2-RFL-T3) and white light source (Olympus, JH40100). Fluorescent images were obtained with GFP, Texas Red, and DAPI filter sets. For direct comparisons within a group, acquisition parameters of each channel were adjusted equally for all images.

Image processing was performed with the Fiji software package [33–35] to combine color channels. For direct comparisons within a group, digital gain of each channel was adjusted equally for all images.

### Axon density quantitation

Axons were counted from nine random regions of interest within the nerve cross section and converted to density with calibrated 20x images. This density was combined with animals with identical genotypes for average values.

### Myelin basic protein Western blot and densitometry

Protein samples were collected from the remaining half of all denervated animals for each genotype. The entire distal stump of the denervated sciatic nerve was flash frozen with liquid nitrogen and stored at -80 until homogenization.

Fresh nerve samples were homogenized with 1.6 mm steel beads (Next Advance, SSB16-RNA) in RIPA buffer (Sigma-Aldrich R1278) containing protease inhibitor (Pierce, 88666). Equal total protein from each lysate was denatured and run on a 4–20% polyacrylamide denaturing gel (BioRad, 456–1093) and transferred to a PVDF membrane (BioRad, 170–4157). Membranes were blotted with primary antibodies rabbit α-myelin basic protein (1:1000; Abcam, 40390) and rabbit α-GAPDH (1:1000; Cell Signaling Technology, 2118S) and secondary antibody donkey α-rabbit, HRP (1:2500; GE Life Sciences NA934V) in 5% milk blocking solution (BioRad 178–6404). Bands were visualized with ECL reagent (GE life sciences, RPN 2106) and digitally imaged on an ImageQuant LAS4000 (GE Life Sciences).

Band density was quantitated using Fiji (see above for Fiji information) and size difference identified myelin basic protein (MBP) versus β-tubulin bands. The ratio of MBP to β-tubulin was calculated for each animal and combined with identical genotypes to give final mean values.
